# Evaluation of infarct size and microvascular reperfusion on angiography and cardiac magnetic resonance in patients with ST-segment elevation myocardial infarction

**DOI:** 10.1186/1532-429X-18-S1-W20

**Published:** 2016-01-27

**Authors:** Justyna Rajewska-Tabor, Aleksander Araszkiewicz, Magorzata Pyda

**Affiliations:** I Cardiology Clinic, University of Medical Sciences in Poznan, Poznan, Poland

## Background

Impaired microvascular reperfusion (no-reflow) and unsuccessful infarct-related artery (IRA) revascularization are associated with worse clinical outcome in patients with ST-segment elevation myocardial infarction (STEMI) treated with primary percutaneous coronary intervention (pPCI). Successful reperfusion can be estimated by epciardial and microvascular flow. Both of them can be evaluated by angiography as Thrombolysis in Myocardial Infarction (TIMI) flow grade and myocardial blush grade (MBG). Moreover microvascular obstruction (MVO) determined by CMR is also a well known predictor of unfavorable clinical outcome after STEMI.

The aim of the study was to evaluate the effect of impaired reperfusion estimated by angiography on microvascular obstruction and infarct size (IS) measured by CMR in STEMI.

## Methods

We examined 85 patients (mean age 59 ± 11 years; 59 males and 26 females) with first STEMI treated with pPCI within 12 hours from symptoms onset. Infarct related artery TIMI flow and MBG were evaluated after pPCI. CMR was performed on the 1.5T system within 96 hours after pPCI. Morphology and function of myocardium was estimated by steady-state free precession (SSFP) sequence. To evaluate the infarct size and MVO, a late gadolinium enhancement (LGE) technique was performed (10-15 min after administration of Gd-DTPA). Infarct size was defined as area above 50% of the maximal signal intensity within LGE (FWMH - full-width half maximum). MVO was defined as the area of absence or hypoenhancement of contrast surrounded by LGE. IS and MVO were determined by planimetry and summation of discs method.

## Results

Revascularization was successful in 41 patients (MBG 3 and TIMI 3) and these patients had preserved ejection fraction (56.3 ± 7.8%) with IS estimated on CMR 16.92 ± 11.35 g and MVO 0.53 ± 1.28 g. The second group, 44 patients with impaired reperfusion (TIMI<3 and/or MBG<3), had similar EF (52.7 ± 11.7%; p = ns), but they had much worse CMR outcomes: IS = 31.7 ± 24.83 g and MVO = 3.42 ± 6.13 g (p = 0.0008 and p = 0.004 respectively). Patients with impaired reperfusion (TIMI<3) had bigger infarct size and MVO on CMR in comparison to patients with normal (TIMI 3) flow in infarct related artery (IRA), (p = 0.007 and p = 0.004 respectively). Similarly, the infarct size and MVO were significantly lower in patients with normal myocardial perfusion (MBG 3) in comparison to patients with worse myocardial perfusion (MBG<3) (p = 0.001 and p = 0.006).

## Conclusions

Impaired reperfusion determined by TIMI and MBG in patients with STEMI influence infarct size and microvascular obstruction evaluated by CMR. The small infarct size and the absence of MVO are associated with successful reperfusion after PCI.

